# Second- and third-generation aromatase inhibitors as first-line endocrine therapy in postmenopausal metastatic breast cancer patients: a pooled analysis of the randomised trials

**DOI:** 10.1038/sj.bjc.6603194

**Published:** 2006-05-30

**Authors:** G Ferretti, E Bria, D Giannarelli, A Felici, P Papaldo, A Fabi, S Di Cosimo, E M Ruggeri, M Milella, M Ciccarese, F L Cecere, A Gelibter, C Nuzzo, F Cognetti, E Terzoli, P Carlini

**Affiliations:** 1 Department of Medical Oncology, Regina Elena Cancer Institute, Rome, Italy; 2 Biostatistics Unit, Regina Elena Cancer Institute, Rome, Italy

**Keywords:** aromatase inhibitors, first-line, endocrine therapy, postmenopausal, metastatic breast cancer, pooled analysis

## Abstract

The purpose of this study was to estimate in all randomised trials the relative risk of overall response rate (ORR), clinical benefit (CB), time to progression (TTP), overall survival (OS), and toxicity of aromatase inhibitors (AI), compared with tamoxifen (Tam) as first-line endocrine therapy in postmenopausal metastatic breast cancer (PMBC) women. Prospective randomised studies were searched through computerised queries of MEDLINE, EMBASE, and the American Society of Clinical Oncology (ASCO) abstract database. Relative risk, 95% confidence interval, and heterogeneity were derived according to the inverse variance and Mantel–Haenszel method and *Q* statistics. Six phase III prospective randomised trials including 2787 women were gathered. A significant advantage in ORR (*P*=0.042), TTP (*P*=0.007), and CB (*P*=0.001) in favour of AI over Tam was detected at the fixed effects model. These results were not significant at the random effects model, owing to the significant heterogeneity. On the contrary, no difference was registered for OS (*P*=0.743) with no significant heterogeneity. Regarding toxicity, Tam caused more frequently thromboembolic events (*P*=0.005) and vaginal bleeding (*P*=0.001) compared with AI. Aromatase inhibitors appear to be superior to Tam as first-line endocrine option in PMBC women. Owing to a component of variability between the six studies analysed, the random effects estimates differed from corresponding fixed ones. Investigators should assess heterogeneity of trial results before deriving summary estimates of treatment effect.

Two-thirds of breast tumours express oestrogen receptor and women having cancers with this characteristic are suitable candidates for endocrine therapy (ET) (Forbes, 1997). Tamoxifen (Tam) has been considered for a long time the drug of choice for postmenopausal women with hormone receptor-positive metastatic breast cancer (MBC). However, Tam is associated with an increased incidence of endometrial cancer and thromboembolic events (TE), and many tumours become resistant to it during treatment (DeFriend *et al*, 1994). Nowadays, the aromatase inhibitors (AI) are considered the treatment of choice for postmenopausal women with newly diagnosed metastases from hormone receptor-positive breast cancer, either in Tam-naïve patients or in those progressing after failing or while on adjuvant Tam. The AI remarkably suppress plasma oestrogen levels in postmenopausal women by inhibiting or inactivating aromatase, the enzyme responsible for the synthesis of oestrogens from androgenic substrates. Third-generation AI (tgAI), including anastrozole, letrozole, and exemestane, have replaced aminoglutethimide in the treatment of postmenopausal patients with MBC because of their considerably lower toxicity. In addition, tgAI have been reported to be more effective and/or less toxic than megestrol acetate (Buzdar *et al*, 1998, 2001a; Dombernowsky *et al*, 1998; Kaufmann *et al*, 2000) and Tam (Bonneterre *et al*, 2000, 2001; Mouridsen *et al*, 2001, 2003; Nabholtz *et al*, 2000, 2003; Paridaens *et al*, 2004). As tgAI have been approved as first-line ET for postmenopausal women with MBC, the issue concerning the optimal sequence of ET has become more challenging. In fact, as AI are more and more frequently used as adjuvant ET, the still open issue concerning the optimal ET sequence will be likely re-challenged in the next future.

A meta-analysis based on patient survival data found a 21% reduction in the risk of death for patients treated with AI (anastrozole, letrozole, and exemestane) compared with those given megestrol (*P*=0.0011) (Messori *et al*, 2000). On the contrary, a pooled analysis has recently suggested that all AI as second-line ET compared with megestrol for postmenopausal MBC patients do not seem to add any significant advantage in terms of overall response rate (ORR) or time to progression (TTP) (Carlini *et al*, 2005). At the present time, the issue as to whether an AI can be more effective than the others remains controversial. Letrozole has been shown to reduce oestrogen levels to a greater degree than the other AI (Boeddinghaus and Dowsett, 2001). Anastrozole has been reported to have greater selectivity for aromatase (Buzdar *et al*, 2001b), but it is still uncertain whether these laboratory findings could affect the clinical decision of preferring one drug instead of another.

The aim of this meta-analysis was to estimate in all published randomised trials the relative risk (RR) of ORR, TTP, clinical benefit (CB), overall survival (OS) and, whenever possible, adverse events of AI compared with Tam as first-line ET in postmenopausal women with MBC.

## MATERIALS AND METHODS

### Outcome definition

We considered the AI as experimental arm and Tam as standard comparator arm. Analysis was conducted in order to find out eventual significant differences in primary and secondary outcomes. Primary outcomes were (1) ORR and (2) TTP. The secondary outcomes were OS, CB, and toxicity, evaluated in at least three trials. In particular, we looked at hot flushes (HF), nausea (N), vomiting (V), TEs, vaginal bleeding (VB), and musculo-skeletal pain (MSP). All calculations were independently performed by two different investigators. The definition of CB (British Breast Group, 1974) was the same across all trials.

### Trial identification criteria

We collected all the prospective randomised trials published as formal papers in peer-reviewed journals or as abstracts in the international congresses proceedings until 31 December, 2004 (Perez Carrion *et al*, 1994; Falkson and Falkson, 1996; Thurlimann *et al*, 1996; Bonneterre *et al*, 2000; Nabholtz *et al*, 2000; Mouridsen *et al*, 2001; Milla-Santos *et al*, 2003; Paridaens *et al*, 2004) (Table 1). In these trials, postmenopausal patients affected by MBC relapsing after adjuvant therapy were randomised to receive AI *vs* standard treatment (Tam). Letters/editorials, studies on AI given as adjuvant/neoadjuvant ET were ruled out.

### Search strategy

Relevant studies were searched through computerised queries of MEDLINE (available from URL: www.ncbi.nlm.nih.gov/PubMed), EMBASE (available from URL: www.embase.com), and the American Society of Clinical Oncology (ASCO) abstract database (available from URL: www.asco.org). Keywords used for research were metastatic breast cancer, aromatase inhibitors, first-line, AI, steroidal, non-steroidal, anastrozole, fadrozole, letrozole, exemestane, formestane, review, metanalysis, meta-analysis, pooled analysis, randomised, phase III, comprehensive review, systematic review, hormonal, and endocrine. Beyond computer browsing, review and original papers were also scanned in the references section to look for missing trials. From each study we obtained (1) rate and number of complete and partial responses, (2) CB (British Breast Group, 1974), (3) median TTP, (4) median OS and (5) rate and number of toxicity events.

### Statistical methods

The log of RR was estimated for each considered end point. Estimated events at 6 months were used when considering TTP and OS. These RRs were combined across the studies, giving weight to the number of events in each of the two treatment groups in each separate study using the Mantel–Haenszel procedure and the inverse variance method; both estimations were performed assuming a fixed effects model (FEM) and a random effects model (REM) (Parmar *et al*, 1998). The heterogeneity between trials was tested with the *Q* statistics, computing the square distance of each study from the combined effect and weighting these values with the inverse of variance of each study (Takkouche *et al*, 1999). The *Q* statistics was then compared with the *χ*^2^ distribution with *k*−1 degrees of freedom, where *k* is the number of studies. All calculations were performed with the Comprehensive Meta-analysis software (version 1.0.23, Biostat, Englewood, NJ, USA) (Bria *et al*, 2005).

## RESULTS

### Selected trials

The eight prospective randomised trials comparing AI *vs* Tam (Perez Carrion *et al*, 1994; Falkson and Falkson, 1996; Thurlimann *et al*, 1996; Bonneterre *et al*, 2000; Nabholtz *et al*, 2000; Mouridsen *et al*, 2001; Milla-Santos *et al*, 2003; Paridaens *et al*, 2004) were conducted between 1994 and 2004, and included 3238 women (Table 1). Globally, 1615 patients were enrolled in the AI arm and 1623 in Tam arm. The median number of patients per trial was 362 (range 80–907). The median follow-up time, when reported, varied much among trials (between 5.1 and 36 months) (Table 1). In the AI arm, TTP ranged between 7.1 and 18 months, and in the Tam arm, between 5.6 and 9.8 months. In the AI arm, the OS range varied between 17.4 and 39.2 months, and in the Tam arm, between 16 and 40 months. In the AI arm, CB ranged between 50 and 83%, and in the Tam arm, between 38 and 75.7%. Hot flushes rate was reported in all trials (3238 patients), N in six trials (Perez Carrion *et al*, 1994; Thurlimann *et al*, 1996; Bonneterre *et al*, 2000; Nabholtz *et al*, 2000; Mouridsen *et al*, 2001; Paridaens *et al*, 2004) (2920 patients), V in five trials (Perez Carrion *et al*, 1994; Thurlimann *et al*, 1996; Bonneterre *et al*, 2000; Nabholtz *et al*, 2000; Paridaens *et al*, 2004) (2012 patients), TE in six trials (Thurlimann *et al*, 1996; Bonneterre *et al*, 2000; Nabholtz *et al*, 2000; Mouridsen *et al*, 2001; Milla-Santos *et al*, 2003; Paridaens *et al*, 2004) (2749 patients), VB in four trials (Bonneterre *et al*, 2000; Nabholtz *et al*, 2000; Milla-Santos *et al*, 2003; Paridaens *et al*, 2004) (1630 patients), and MSP in four trials (Bonneterre *et al*, 2000; Nabholtz *et al*, 2000; Milla-Santos *et al*, 2003; Paridaens *et al*, 2004) (2299 patients). The primary and secondary end points are indicated in Table 1. All arms within each trial were well balanced for pretreatment characteristics of the patients.

For our meta-analysis, we selected only the phase III studies published as original papers in peer-review journals. These studies (Perez Carrion *et al*, 1994; Thurlimann *et al*, 1996; Bonneterre *et al*, 2000; Nabholtz *et al*, 2000; Mouridsen *et al*, 2001; Milla-Santos *et al*, 2003) globally included 2787 women. The trial by Falkson and Falkson (1996), which was a randomised phase II study, was excluded. The study by Paridaens *et al* (2004), which was a large randomised phase III trial presented at the 2004 ASCO annual meeting but published exclusively in the abstract format, was included only in the comparisons (efficacy and toxicity) between tgAI *vs* Tam.

### Combined analysis

All outcomes and their statistical significance are listed in Table 2. Risk ratios have to be interpreted as follows: regarding ORR and CB, RR more than 1.0 favours AI, whereas RR less than 1.0 favours Tam; concerning TTP and OS, RR less than 1.0 favours AI, whereas RR more than 1.0 favours Tam (event-based analysis, see Statistical methods).

**Figure 1:**
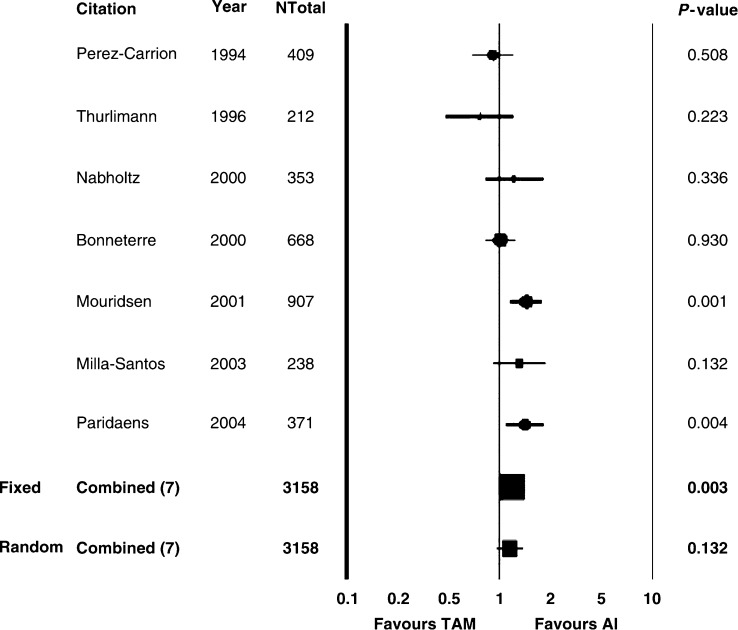
Aromatase inhibitors *vs* tamoxifen: ORR. AI: aromatase inhibitors; TAM: tamoxifen; Ntot: total number of patients; RR: relative risk; Fixed: fixed effects model; Random: random effects model; ORR: overall response rate.

**Figure 2:**
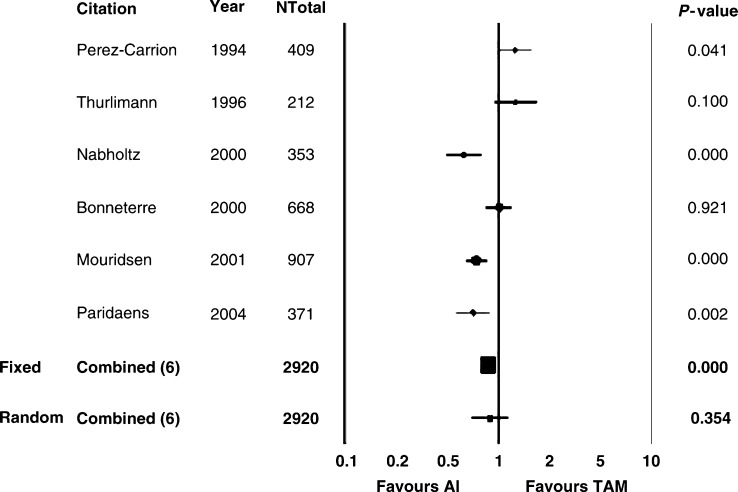
Aromatase inhibitors *vs* tamoxifen: TTP. AI: aromatase inhibitors; TAM: tamoxifen; Ntot: total number of patients; RR: relative risk; Fixed: fixed effects model; Random: random effects model.

We compared AI *vs* Tam in the overall population (2787 patients), using the FEM first. A significant advantage in ORR in favour of AI over Tam was detected (RR=1.13, 95% confidence interval (CI) 1.00–1.28, *P*=0.042) (Table 2 and Figure 1). The same impact in favour of AI was seen for TTP (2549 patients), where RR was 0.88 (95% CI 0.80–0.96, *P*=0.007) (Table 2 and Figure 2). Moreover, concerning CB, a statistically significant advantage in favour of AI compared with Tam was observed (RR 1.11, 95% CI 1.04–1.19, *P*=0.001). On the contrary, no significant difference was registered for OS (RR 0.97, 95% CI 0.79–1.18, *P*=0.743) (Table 2). A significant heterogeneity for ORR (0.03), TTP (<0.0001), and CB (<0.0001) was registered using the FEM (Table 2). At the REM, the significant improvement in ORR, TTP, and CB in favour of AI over Tam was not confirmed. By contrast, no significant heterogeneity was observed regarding OS estimates (Table 2).

Comparing non-steroidal AI (nsAI) *vs* Tam using the FEM, a significant advantage in ORR favouring nsAI *vs* Tam was registered (RR=1.23, 95% CI 1.07–1.42, *P*=0.003) (Table 3). The same advantage in favour of nsAI was seen for TTP, where RR was 0.77 (95% CI 0.69–0.86, *P*=<0.0001). A statistically significant result was observed also for CB in favour of nsAI over Tam (RR 1.21, 95% CI 1.12–1.31, *P*<0.0001). No significant difference was registered for OS (RR 0.94, 95% CI 0.75–1.78, *P*=0.599) (Table 3). A significant heterogeneity for TTP (0.002) and CB (0.005) was registered at the FEM (Table 3). When the REM was used, only the improvement in CB was confirmed. No significant heterogeneity was observed regarding OS estimates (Table 3).

Comparing tgAI *vs* Tam by the fixed effects estimate (FEM), a significant advantage in ORR favouring tgAI *vs* Tam was observed (RR=1.28, 95% CI 1.13–1.44, *P*<0.0001) (Table 4). The same advantage in favour of tgAI was seen for TTP, where RR was 0.76 (95% CI 0.69–0.84, *P*<0.0001). A statistically significant advantage was observed also for CB in favour of tgAI over Tam (RR 1.23, 95% CI 1.14–1.32, *P*<0.0001). No significant difference was registered for OS (RR 0.93, 95% CI 0.76–1.15, *P*=0.529) (Table 4). A significant heterogeneity for TTP (0.004) and CB (0.008) was registered at the FEM (Table 4). Using the REM, the significant improvement in TTP and CB in favour of AI over Tam was confirmed. No significant heterogeneity was observed regarding OS estimates (Table 4).

**Figure 3:**
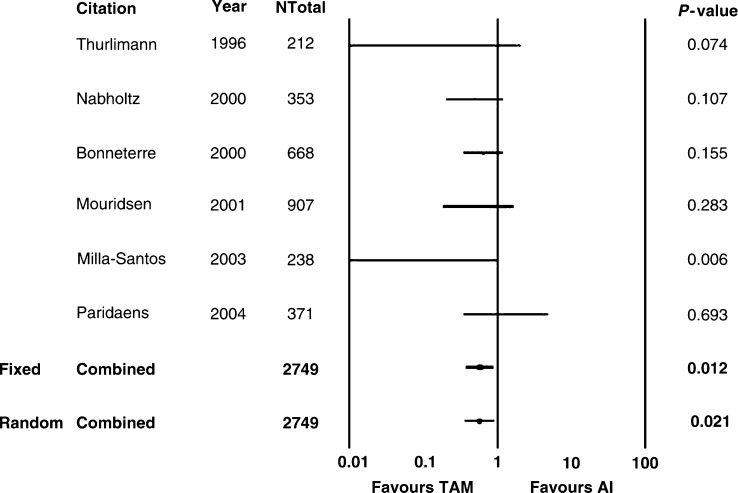
Aromatase inhibitors *vs* tamoxifen: TEs. AI: aromatase inhibitors; TAM: tamoxifen; Ntot: total number of patients; RR: relative risk; Fixed: fixed effects model; Random: random effects model.

**Figure 4:**
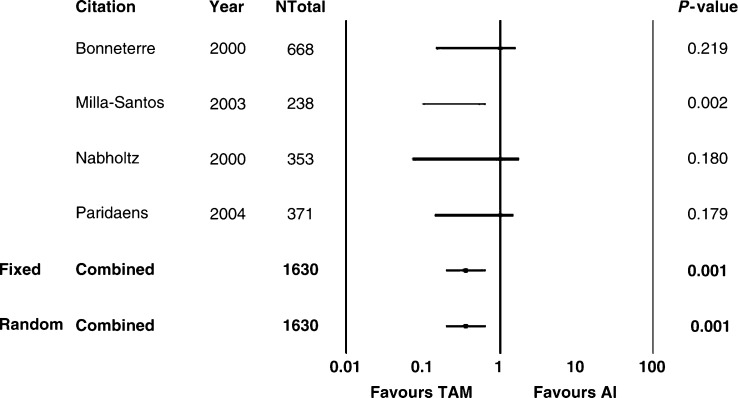
Aromatase inhibitors *vs* tamoxifen: VB. AI: aromatase inhibitors; TAM: tamoxifen; Ntot: total number of patients; RR: relative risk; Fixed: fixed effects model; Random: random effects model.

Regarding toxicity at the FEM, Tam caused more frequently TE (RR 0.53, 95% CI 0.34–0.82, *P*=0.005) and VB (RR 0.33, 95% CI 0.17–0.65, *P*=0.001) (Table 5 and Figures 3 and 4). No significant difference was observed in HF (*P*=0.171), N (*P*=0.547), V (*P*=0.686), and MSP (*P*=0.598) (Table 5). Similar results were observed comparing nsAI *vs* Tam (Table 6) or tgAI *vs* Tam (Table 7). Excluding HF, no significant heterogeneity was registered concerning toxicity, in particular with respect to TE and VB (Table 4). Regarding HF reported using nsAI or tgAI *vs* Tam, the findings reported by the FEM were confirmed at the REM.

## DISCUSSION

The AI have been reported to be superior to Tam as initial therapy for postmenopausal women with MBC (Nabholtz *et al*, 2000; Bonneterre *et al*, 2001; Mouridsen *et al*, 2001, 2003; Paridaens *et al*, 2004). Our analysis of abstracted data coming from six trials using AI as first-line endocrine option in comparison with Tam in postmenopausal women with MBC detected a statistically significant improvement in ORR, CB, and TTP in favour of AI over Tam (FEM). These results were not significant at the REM, owing to the strongly significant heterogeneity (Table 2 and Figure 1 and 2). Stratifying for type of AI (steroidal and non-steroidal), a significant difference (FEM) in ORR, CB, and TTP in favour of nsAI over Tam was observed as well. At the REM, only the improvement in CB was confirmed, whereas the advantage in TTP lost its significance (Table 3). Fadrozole (Tominaga *et al*, 2003) and formestane (Vorobiof *et al*, 1999) have been shown to be inferior to letrozole and anastrozole, respectively. For this reason, we excluded fadrozole and formestane studies from the analysis of AI *vs* Tam, in order to make the differences greater. Comparing tgAI *vs* Tam, a significant difference (FEM) in ORR, CB, and TTP in favour of tgAI over Tam was observed. It must be highlighted that the results concerning TTP and CB maintained their significance at the REM, despite the significant heterogeneity (Table 4). By contrast, there was no clear evidence of benefit in OS comparing AI with Tam, without significant heterogeneity. However, OS estimation in MBC could be affected by several factors, such as prior or subsequent chemotherapy or hormonal treatments or crossover design, and TTP might be regarded as the most sensitive parameter to assess efficacy of a new drug, especially when TTP increase is associated with ORR increase (Di Leo and Bleiberg, 2003). Finally, owing to the above-mentioned significant heterogeneity, any definitive conclusion about ORR, TTP, and CB cannot be conclusively affirmed.

The availability of results using both the REM and FEM in electronic publications could represent a temptation to select the model that better supports the authors' hypothesis, introducing a potential source of bias in the interpretation of meta-analysis results. Heterogeneity is an important issue in meta-analyses. When there is no component of variability between studies, the results of methods based on FEM or REM are essentially identical and both methods yield similar point estimates (Greenland, 1987; Berlin *et al*, 1989). When there is heterogeneity between studies, fixed effects standard errors often suggest inappropriate precision and the CI for a summary estimate of effects size will be wider when the random effect is used. Thus, as heterogeneity is incorporated directly into random effects summary estimates and their standard errors, it is not surprising that random effects estimates sometimes differ from corresponding fixed effects ones (Engels *et al*, 2000). The overall effect of heterogeneity could be to make most random effects estimates less significant than the corresponding fixed effects estimates. The REM assumption that trials included in a meta-analysis are a random sample from a large population of trials would seem to be less defendable in the context of systematic reviews, which, by definition, aim at the inclusion of all published and unpublished trials considered as the population from which this assumption is being inferred (Villar *et al*, 2001). The REM inference concerning parameters of a population larger than those trials available, including trials that may be carried out later, does not seem relevant in the context of meta-analyses conducted as part of systematic reviews of randomised controlled trials. Such reviews explore mostly the question as to whether the treatment can produce benefit on average in the studies at hand (Bailey, 1987). Finally, several authors present arguments for and against routine use of models based on REM or FEMs in meta-analysis (Greenland, 1987; Peto, 1987; Berlin *et al*, 1989; Thompson and Pocock, 1991), but there is no clear consensus yet.

Heterogeneity is not only statistical *per se*, but is also closely related to the study design of the systematic review, the nature of the trials included, the intended extrapolation of the results, and the clinical relevance of the observed differences. Therefore, systematic reviews might locate all available trials (even if unpublished) and make extensive efforts to include them, similar to the efforts typically made to reduce loss to follow-up in clinical trials. In the presence of statistical heterogeneity, the main focus of a meta-analysis should be on trying to understand clinical sources of heterogeneity. The significant heterogeneity, which proves trial interaction, could easily depend on different patients selection (i.e., differences in the characteristics of study subjects, such as their mean age and the severity of illness, positive or unknown receptor status, node-negative or node-positive disease), different trial designs, different rates of loss to follow-up, different interventions (dose or duration of treatment), or outcome measures. For example, the study by Mouridsen *et al* (2001) had almost twice as many patients with prior Tam therapy than the study by Bonneterre *et al* (2000) and Nabholtz *et al* (2000) (22 *vs* 7.6 *vs* 11.6% total) (Copur *et al*, 2001). In four studies (Falkson and Falkson, 1996; Thurlimann *et al*, 1996; Mouridsen *et al*, 2001; Paridaens *et al*, 2004), more than 30% of the patients had previously received chemotherapy, whereas in other three studies (Falkson and Falkson, 1996; Bonneterre *et al*, 2000; Nabholtz *et al*, 2000), this percentage ranged between 5 and 21%. In five trials (Thurlimann *et al*, 1996; Nabholtz *et al*, 2000; Mouridsen *et al*, 2001; Milla-Santos *et al*, 2003; Paridaens *et al*, 2004), more than 65% of the patients had hormone receptor-positive tumours compared with only 43% in the three remainder studies (Perez Carrion *et al*, 1994; Falkson and Falkson, 1996; Bonneterre *et al*, 2000). Conversely, most of the patients in the study by Bonneterre *et al* (2000) (54.4% in the anastrozole group and 55.8% in the tamoxifen group) had tumours with unknown hormone receptors, whereas Nabholtz *et al* (2000) reported only 11.1 and 11% and Mouridsen *et al* (2001) 34 and 33%, respectively (Costa and Kaufmann, 2001). Almost 50% of the patients in the study by Bonneterre *et al* (2000) had advanced-stage disease at presentation and therefore had received no prior treatment. This is 20% more than for the women in the North American study (Nabholtz *et al*, 2000) and 25% more than for patients in the Mouridsen *et al*'s (2001) study. The group of patients in the study by Bonneterre *et al* (2000) had 88% measurable disease, compared with 68–76% measurable disease as in the North American patients (Nabholtz *et al*, 2000). More importantly, in Nabholtz *et al*'s (2000) study, the tamoxifen group had an excess of patients with liver disease (16.5%) (Tonkin, 2001) compared with Bonneterre *et al*'s (2000) study (9.5%) and Mouridsen *et al*'s (2001) study (viscera only 13%).

Concerning toxicity, we chose the most frequently reported side effects (at least in three trials), in order to obtain reliable comparisons between AI and Tam. Thus, toxicity was not evaluated completely, and, for this reason, the reported results must be interpreted cautiously. The analysed adverse events were HF, N, V, TEs, VB, and MSP. Thromboembolic phenomena and VB were observed more commonly in Tam-treated patients compared with those receiving AI (*P*=0.01 and *P*=0.001, respectively), without significant heterogeneity. No significant difference was present between AI *vs* Tam in terms of HF, N, V, and MSP, without significant heterogeneity. Our findings resembled those already reported in the literature, TE and VB being more frequent with the use of Tam.

Based on the presented results, AI appear to be superior to Tam as first-line endocrine option in postmenopausal women with MBC, as a significant benefit in terms of ORR, CB, and TTP was observed in favour of AI over Tam with fixed effects estimates. Owing to a component of variability between the six studies analysed, the random effects estimates differed from corresponding fixed ones. Concerning the toxicity profile, AI, as expected, caused less thromboembolic phenomena and VB than Tam. Considering our findings, it would be crucial to analyse the cost–utility balance of AI compared with Tam as first-line endocrine treatment of MBC. It has been recently reported (Dranitsaris *et al*, 2003; Marchetti *et al*, 2004) that tgAI represent an economically acceptable alternative to Tam. Although investigators should assess heterogeneity of trial results before deriving summary estimates of treatment effect, we think that these findings might be taken into account in the oncology practice during the clinical decision-making process.

## Figures and Tables

**Table 1:** Characteristics of the studies

**RCTs**	**Year**	**Pts**	**Arms**	**Primary/secondary end points**	**Median follow-up (months)**	**Key results**
Perez Carrion	1994	409	FOR *vs* TAM	ORR (UICC)/TTP, TTF, OS, safety	NR	No difference in ORR and OS, longer TTP and TTF in the TAM arm
Thurlimann	1996	212	FDZ *vs* TAM	ORR (WHO)/TTF, OS, safety	36	Not double-blinded, no difference in ORR and OS, longer TTF in the TAM arm
Falkson	1996	80	FDZ *vs* TAM	ORR (ECOG)/TTF, OS, safety	5.1	No difference in ORR, TTF, and OS
Nabholtz	2000	353	ANA *vs* TAM	ORR (UICC), TTP, safety/TTF, CB, OS	17.7	No difference in ORR, longer TTP, and higher CB in the ANA arm
Bonneterre	2000	668	ANA *vs* TAM	ORR (UICC), TTP, safety/TTF, CB, OS	19	No difference in ORR, TTP, and CB
Mouridsen	2001	907	LTZ *vs* TAM	TTP/ORR (UICC), TTF, CB, OS, safety	32	Longer TTP, higher ORR and CB in the LTZ arm
Milla Santos	2003	238	ANA *vs* TAM	CB, ORR (WHO), TTP, OS, safety	13.3	Longer TTP and OS, higher CB, in the ANA arm
Paridaens	2004	371	EXE *vs* TAM	PFS/OS, safety	NR	Longer PFS, higher ORR in the EXE arm
Total		3238				

ANA=anastrozole; CB=clinical benefit; EXE=exemestane; FDZ=fadrozole; FOR=formestane; LTZ=letrozole; NR=not reported; ORR=overall response rate; OS=overall survival; PFS=progression-free survival; pts=patients; RCTs=randomised clinical trials; TAM=tamoxifen; TTF=time to treatment failure; TTP=time to progression.

**Table 2:** Efficacy: aromatase inhibitors *vs* tamoxifen (FEM and REM)

	**RCTs**	**Pts**	**RR (FEM)**	**RR (REM)**	**95% CI (FEM)**	**95% CI (REM)**	***P* (FEM)**	**Het.**	***P* (REM)**
ORR	6	2787	1.13	1.11	1.00, 1.28	0.89, 1.37	0.042	0.03	0.343
TTP	5	2549	0.88	0.92	0.80, 0.96	0.68, 1.26	0.007	<0.0001	0.637
CB	6	2787	1.11	1.13	1.04, 1.19	0.96, 1.33	0.001	<0.0001	0.123
OS	6	2787	0.97		0.79, 1.18		0.743	0.98	

CI=confidence intervals; FEM=fixed effects model; Het=heterogeneity; ORR=overall response rate; OS=overall survival; Pts=patients; RCTs=randomised clinical trials; REM=random effects model; RR=relative risk ratio; TTP=time to progression.

**Table 3:** Efficacy: non-steroidal aromatase inhibitors *vs* tamoxifen (FEM and REM)

	**RCTs**	**Pts**	**RR (FEM)**	**RR (REM)**	**95% CI (FEM)**	**95% CI (REM)**	***P* (FEM)**	**Het.**	***P* (REM)**
ORR	4	2166	1.23		1.07, 1.42		0.003	0.10	
TTP	3	1928	0.77	0.76	0.69, 0.86	0.55, 1.05	<0.0001	0.002	0.098
CB	4	2166	1.21	1.25	1.12, 1.31	1.03, 1.50	<0.0001	0.005	0.018
OS	4	2166	0.94		0.75, 1.78		0.599	0.94	

CI=confidence intervals; FEM: fixed effects model; Het=heterogeneity; ORR=overall response rate; OS=overall survival; Pts=patients; RCTs=randomised clinical trials; REM=random effects model; RR=relative risk ratio; TTP=time to progression.

**Table 4:** Efficacy: third-generation aromatase inhibitors *vs* tamoxifen (FEM and REM)

	**RCTs**	**Pts**	**RR (FEM)**	**RR (REM)**	**95% CI (FEM)**	**95% CI (REM)**	***P* (FEM)**	**Het.**	***P* (REM)**
ORR	5	2537	1.28		1.13, 1.44		<0.0001	0.12	
TTP	4	2299	0.76	0.74	0.69, 0.84	0.58, 0.94	<0.0001	0.004	0.015
CB	5	2537	1.23	1.26	1.14, 1.32	1.09, 1.46	<0.0001	0.008	0.0002
OS	5	2537	0.93		0.76, 1.15		0.529	0.98	

CI=confidence intervals; FEM=fixed effects model; Het=heterogeneity; ORR=overall response rate; OS=overall survival; Pts=patients; RCTs=randomised clinical trials; REM=random effects model; RR=relative risk ratio; TTP=time to progression.

**Table 5:** Toxicity: aromatase inhibitors *vs* tamoxifen (FEM)

	**RCTs**	**Pts**	**RR**	**95% CI**	***P***	**Het.**
HF	6	2787	1.11	0.95, 1.30	0.171	0.06
Nausea	5	2549	0.94	0.78, 1.13	0.547	0.67
Vomiting	4	1642	1.08	0.72, 1.62	0.686	0.49
TE	5	2378	0.53	0.34, 0.82	0.005	0.42
VB	3	1259	0.33	0.17, 0.65	0.001	0.71
MSP	3	1928	1.05	0.87, 1.26	0.598	0.79

CI=confidence intervals; FEM=fixed effects model; Het=heterogeneity; HF=hot flushes; MSP=muscolo-skeletal pain; ORR=overall response rate; OS=overall survival; Pts=patients; RCTs=randomised clinical trials; REM=random effects model; RR=relative risk ratio; TE: thromboembolic events; VB: vaginal bleeding.

**Table 6:** Toxicity: non-steroidal aromatase inhibitors *vs* tamoxifen (FEM and REM)

	**RCTs**	**Pts**	**RR (FEM)**	**RR (REM)**	**95% CI (FEM)**	**95% CI (REM)**	***P* (FEM)**	**Het.**	***P* (REM)**
HF	4	2166	1.13	1.01	0.95, 1.33	0.69, 1.43	0.160	0.01	0.95
Nausea	3	1928	0.94		0.78, 1.13		0.530	0.85	
Vomiting	2	1021	1.09		0.71, 1.68		0.692	0.52	
TE	4	2166	0.55		0.35, 0.86		0.009	0.43	
VB	3	1259	0.33		0.17, 0.65		0.001	0.71	
MSP	3	1928	1.05		0.87, 1.26		0.598	0.79	

CI=confidence intervals; FEM=fixed effects model; Het=heterogeneity; HF=hot flushes; MSP=muscolo-skeletal pain; ORR=overall response rate; OS=overall survival; Pts=patients; RCTs=randomised clinical trials; REM=random effects model; RR=relative risk ratio; TE=thromboembolic events; VB=vaginal bleeding.

**Table 7:** Toxicity: third-generation aromatase inhibitors *vs* tamoxifen (FEM and REM)

	**RCTs**	**Pts**	**RR (FEM)**	**RR (REM)**	**95% CI (FEM)**	**95% CI (REM)**	***P* (FEM)**	**Het.**	***P* (REM)**
HF	5	2537	1.07	1.01	0.93, 1.23	0.76, 1.35	0.352	0.02	0.91
Nausea	4	2299	0.92		0.78, 1.09		0.381	0.91	
Vomiting	3	1392	1.14		0.78, 1.64		0.487	0.75	
TE	5	2537	0.60		0.39, 0.92		0.021	0.37	
VB	4	1630	0.36		0.20, 0.64		0.0006	0.82	
MSP	4	2299	1.01		0.87, 1.18		0.823	0.83	

CI=confidence intervals; FEM=fixed effects model; Het=heterogeneity; HF=hot flushes; MSP=muscolo-skeletal pain; ORR=overall response rate; OS=overall survival; Pts=patients; RCTs=randomised clinical trials; REM=random effects model; RR=relative risk ratio; TE=thromboembolic events; VB=vaginal bleeding.
